# Commentary: Case Report: Experience of a rare case of primary acute mast cell leukemia with FGFR1 gene rearrangement

**DOI:** 10.3389/fonc.2026.2001012

**Published:** 2026-10-19

**Authors:** Huizhen Du, Congcong Geng, Na Zhu, Zhao Li, Yalu Liu, Chunyan Liu, Jing Niu, Qian Wang, Hongwei Ren

**Affiliations:** 1 Department of Clinical Laboratory, Qilu Hospital (Qingdao), Cheeloo College of Medicine, Shandong University, Qingdao, Shandong, China; 2 Department of hematology, Qilu Hospital (Qingdao), Cheeloo College of Medicine, Shandong University, Qingdao, Shandong, China; 3 Department of Traditional Chinese Medicine, School of Basic Medicine, Qingdao University, Qingdao, China; 4 Department of Clinical Psychology, Xuzhou Keshubao Children’s Hospital, Xuzhou, Jiangsu, China; 5 Department of Clinical Laboratory, Qilu Hospital of Shandong University, Jinan, China; 6 Centre for Haematology, Department of Immunology and Inflammation, Imperial College London, London, United Kingdom

**Keywords:** CD117, diagnostic discordance, FGFR1 rearrangement, flow cytometry, immunophenotyping, mast cell

## Introduction

We wish to highlight a diagnostic lesson embedded in the previously published case described as primary acute mast-cell leukemia with an FGFR1 rearrangement (1). The molecular association itself has already been reported and is not presented here as a new observation. In our view, the case illustrates both a practical problem in lineage assignment when flow cytometry and tissue-based assessment appear discordant and a broader classification question raised by the presence of an FGFR1 rearrangement.

## The diagnostic discordance

In the original report, bone marrow flow cytometry identified 17.54% abnormal mast cells and a second large aberrant population comprising 63.5% of nucleated cells. This second population was reported as expressing strong CD33, weak CD11b and CD2, while lacking CD13, CD34, CD117 and CD203c; flow cytometry therefore could not confidently determine its cellular origin (1). The published flow-cytometric plot may not, on retrospective visual inspection alone, permit an unequivocal distinction between complete CD117 negativity and very dim CD117 expression. Bone marrow biopsy immunohistochemistry was reported to show abnormal cells expressing CD117, CD25, CD2 and tryptase (1). The original report did not describe a distinct dual (dim/strong) CD117 staining pattern on immunohistochemistry. We therefore interpret the central diagnostic issue as cross-platform discordance in lineage assignment rather than as proof of absolute CD117 loss in a defined clone.

## Why CD117 should not be interpreted alone

CD117 is a highly useful marker for mast-cell identification, but mast-cell immunophenotyping is vulnerable to biological and technical variation. A recent review of flow-cytometric pitfalls emphasizes that no single antigen is completely specific and that accurate mast-cell identification requires a multiparametric approach incorporating marker combinations, gating strategy and awareness of interfering populations (2). We suggest that, in a case with an unusual immature phenotype, reported absence or very dim expression of CD117 or CD203c within a flow-cytometric population should prompt reconciliation with morphology, histology and complementary immunophenotyping rather than immediate exclusion of mast-cell differentiation. The original publication also showed CD68 staining, but CD68 is not used in this Commentary as independent evidence of mast-cell lineage; our interpretation instead relies on the published morphology and the more lineage-informative mast-cell-associated findings, including tryptase (1).

## Implications for FGFR1-rearranged disease

The FGFR1 rearrangement adds an important classification issue. Current WHO and International Consensus Classification frameworks recognize FGFR1-rearranged disease within the genetically defined spectrum of myeloid/lymphoid neoplasms with tyrosine-kinase gene fusions (3, 4). These neoplasms may show heterogeneous myeloid and/or lymphoid manifestations during the disease course, and eosinophilia, although characteristic in many cases, is not obligatory. The original report documented leukocytosis, splenomegaly and mildly FDG-avid enlarged hepatotactic and retroperitoneal lymph nodes, but did not report a lymph-node biopsy or provide an eosinophil count in the main article (1). It also stated that marrow morphology showed no dyserythropoiesis, ring sideroblasts, multinuclearity or myeloid blasts and on that basis excluded an associated hematologic neoplasm (1). However, in the setting of an FGFR1 rearrangement, the published findings also raise the alternative possibility that the prominent mast-cell proliferation represented a manifestation of an FGFR1-rearranged myeloid/lymphoid neoplasm rather than an independent mast-cell neoplasm carrying an incidental FGFR1 rearrangement. The available published data do not resolve these alternatives. The reported complex karyotype included dic(8;11)(p12;q12); involvement of 8p11–12 is cytogenetically compatible with the FGFR1 rearrangement subsequently demonstrated by break-apart FISH, although the specific fusion partner was not established (1).

## Limitations and practical lesson

No additional patient data are introduced in this commentary. The original report stated that Illumina high-throughput sequencing showed a wild-type KIT sequence and identified SETD2 and GNB1 mutations, whereas the FGFR1 rearrangement was demonstrated by FISH (1). Because the published report does not provide sufficient assay-level detail to verify coverage of individual non-D816V KIT variants, absence of KIT D816V alone should not be interpreted as excluding all activating KIT mutations; non-D816V variants including K509I, N819Y and I817V have been reported in mast-cell disease (5). Likewise, the original publication did not report the CD117 antibody clone used for immunohistochemistry, isotype-control confirmation of the flow-cytometric CD117 assessment, a separate RNA-based assay defining the FGFR1 fusion transcript, a lymph-node biopsy, or sufficient information in the main article to characterize eosinophilia. The flow-cytometric population reported as CD117-negative was not separately sorted for molecular analysis; therefore, the published data cannot establish clonal identity among the phenotypically different populations or distinguish definitively between primary mast-cell leukemia with an FGFR1 rearrangement and an FGFR1-rearranged myeloid/lymphoid neoplasm with prominent mast-cell differentiation. Our practical conclusion is therefore deliberately cautious: when morphology, tissue immunophenotyping, flow cytometry and a defining genomic abnormality appear discordant, lineage and disease classification should be based on integrated review rather than on CD117 or any single platform alone.
